# Examining the role of maternal religiosity in offspring mental health using latent class analysis in a UK prospective cohort study

**DOI:** 10.1017/S003329172300079X

**Published:** 2023-11

**Authors:** Isaac Halstead, Jon Heron, Connie Svob, Carol Joinson

**Affiliations:** 1 The Centre for Academic Child Health, Population Health Sciences, Bristol Medical School, University of Bristol, Bristol, Gloucestershire, BS8 2BN, UK; 2 Population Health Sciences, Bristol Medical School, University of Bristol, Bristol, Gloucestershire, BS8 2BN, UK; 3 Department of Psychiatry, Vagelos College of Physicians and Surgeons, New York, NY, USA; 4 Department of Epidemiology, Mailman School of Public Health, Columbia University, New York, NY, USA; 5 Division of Child & Adolescent Psychiatry, New York State Psychiatric Institute, New York, NY, USA

**Keywords:** ALSPAC, latent class analysis, mental health, religion, religious belief

## Abstract

**Background:**

Previous research has examined the role of parental religious belief in offspring mental health, but has revealed inconsistent results, and suffered from a number of limitations. The aim of this study is to examine the prospective relationship between maternal religiosity and offspring mental health and psychosocial outcomes.

**Methods:**

We used latent classes of religious belief (Highly religious, Moderately religious, Agnostic, Atheist) in mothers from the Avon Longitudinal Study of Parents and Children from 1990, and examined their association with parent-reported mental health outcomes and self-reported psychosocial outcomes in their children at age 7–8 (*n* = 6079 for mental health outcomes and *n* = 5235 for psychosocial outcomes). We used inverse probability weighted multivariable logistic regression analysis adjusted for maternal mental health, adverse childhood experience, and socioeconomic variables.

**Results:**

There was evidence for a greater risk of internalising problems among the offspring of the Highly religious and Moderately religious classes [e.g. for depression; OR 1.40. 95% CI (1.07–1.85), OR 1.48, 95% CI (1.17–1.87)], and greater risk of externalising problems in the offspring of the Atheist class [e.g. for ADHD; OR 1.41, 95% CI (1.08–1.85)], compared to the offspring of the Agnostic class.

**Conclusions:**

These novel findings provide evidence associations between maternal religiosity and offspring mental health differ when examined using a person-centred approach, compared to the previously used variable-centred approaches. Our findings also suggest that differences may exist in the relationship between religious (non)belief and mental health variables when comparing the UK and US.

## Introduction

Childhood mental health problems place a strain on both the child and their family (Farrell & Barrett, 2007; Houtrow & Okumura, 2011). Parental factors such as socioeconomic status, parenting style, and parental mental health are important predictors of mental health outcomes in children (Bøe et al., 2014; Leinonen, Solantaus, & Punamäki, 2003; Manning & Gregoire, 2009; Melchior & van der Waerden, 2016). There is also some evidence for parental religiosity playing a role in offspring mental health, but the limited research that exists has found inconsistent associations (Bartkowski, Xu, & Levin, 2008; Schottenbauer, Spernak, & Hellstrom, 2007; Svob et al., 2018). We define religiosity as a combination of beliefs, behaviours and rituals related to a higher or divine power (adapted from Koenig, 2009). Some studies that examined the relationship between parental religiosity and child mental health have found evidence for associations between parent religiosity and child internalising (Schottenbauer et al., 2007), such as depression and anxiety. Others have found associations with externalising problems (Bartkowski et al., 2008) such as hyperactivity, conduct disorder and oppositional defiant disorder (ODD). One study has also found an association with lower suicidal ideation (Svob et al., 2018). However, other studies have found a mixture of positive or no relationship (van der Jagt-Jelsma et al., 2015, 2017), or indirect relationships with mental health, through parenting factors (Kim-Spoon, Longo, & McCullough, 2012). Overall, there is stronger evidence for an association between parental religiosity and internalising symptoms than externalising symptoms, but previous research has only focussed on one or two mental health outcomes per study. Our intention is to simultaneously explore a range of both internalising and externalising outcomes, which may provide evidence for differential relationships between specific mental health outcomes and parental religious belief.

Some inter-study variability may be attributed to small sample sizes (e.g. Kim, McCullough, & Cicchetti, 2009; Kim-Spoon et al., 2012; Svob et al., 2018; van der Jagt-Jelsma et al., 2017; Varon & Riley, 1999 all have less than 600 participants) and inadequate adjustment for confounders (e.g. Kim-Spoon et al., 2012; Schottenbauer et al., 2007; van der Jagt-Jelsma et al., 2017, which use no confounds, only parenting variables, and marital status and socioeconomic status, respectively). These studies are also limited due to study design – of all the studies mentioned that examine parental religiosity and offspring mental health, only three use a longitudinal cohort study design, (the rest being cross-sectional) and none of these examined parental belief before the birth of the child.

Parental mental health is a potentially important confounder of the relationship between parental religiosity and child mental health. A large body of evidence indicates that parental mental health is a strong and consistent predictor of offspring mental health outcomes (Bould et al., 2015; Dean et al., 2010; Jacobs, Miller, Wickramaratne, Gameroff, & Weissman, 2012; Jacobs, Talati, Wickramaratne, & Warner, 2015), and an individual's religiosity is consistently, positively related to their own mental health in US samples (Braam & Koenig, 2019; Koenig, 2009). Parental socioeconomic position (SEP) is also a plausible confounder of the relationship between parental religiosity and offspring mental health. Higher parental SEP is associated with better mental and physical health in offspring (Cohen, Yoon, & Johnstone, 2009; Lemstra et al., 2008; Vukojević et al., 2017). There is also evidence for a relationship (albeit an inconsistent one) between socioeconomic variables and religiosity (Brandt & Henry, 2012; Heaton, 2013; Horowitz & Garber, 2003; Mueller & Johnson, 1975; Schieman, 2010; Schwadel, 2015; Storm, 2017; Thompson, Thomas, & Head, 2012), which was also identified in Avon Longitudinal Study of Parents and Children (ALSPAC) (Halstead, Heron, & Joinson, 2022; Major-Smith et al., 2023). Adverse childhood experiences (ACEs) are also associated with subsequent religious struggles (e.g. feelings of abandonment by God) such as after the death of a loved one or life-threatening events (McCormick, Carroll, Sims, & Currier, 2017), but also a desire to connect to a higher power (Santoro, Suchday, Benkhoukha, Ramanayake, & Kapur, 2016), and a parent's ACEs are associated with offspring mental health outcomes (Schickedanz, Halfon, Sastry, & Chung, 2018). Finally, greater parental age is simultaneously related to greater religiosity (Schwadel, 2011), and better child mental health (albeit inconsistently) (Zondervan-Zwijnenburg et al., 2020), and should be included as a confounder.

Furthermore, previous research has been dominated by US samples. There is evidence for differences in the relationship between religious belief and mental health in the US compared to other countries, such as the UK (United Kingdom), Korea, Spain, The Netherlands, Slovenia, Estonia, Portugal, and Chile (King et al., 2013; Leurent et al., 2013; Lewis, Maltby, & Day, 2005; Park, Hong, Park, & Cho, 2012). Epidemiological and psychological studies that examine parental religious belief and offspring mental health have also almost exclusively used single item measures of religiosity – attendance at a place of worship, or the importance of religion in their lives. While commonly used in the religious belief literature, church attendance functions poorly when differentiating Atheists and Agnostics, which are both unlikely to attend church, barring weddings, funerals etc. Additionally, the importance of church attendance may also differ between religious denominations. Consequently, use of these items may be artificially constraining the variety of distinct kinds of religious (non)belief and conceal their true relationships to outcome variables. Furthermore, given the nature of religiosity, which consists of a range of beliefs and practices, it is likely to be a multidimensional construct, and unlikely to be comprehensively measured with a single dimension or item.

Religiosity also relates to a variety of childhood psychosocial outcomes, such as higher self-worth (Top, Chadwick, & McClendon, 2003), academic achievement/scholastic competence (Jeynes, 2003; McKune & Hoffmann, 2009), and lower antisocial behaviours (Adamczyk, 2012; Laird, Marks, & Marrero, 2011; Munir & Malik, 2020). However, the role of parental religiosity has not been extensively examined (Abar, Carter, & Winsler, 2009; Bartkowski et al., 2008; Regnerus, 2003), with a mixture of positive, negative, and no associations.

The present study, based on data from a large birth cohort, the ALSPAC, examines the prospective relationship between maternal religiosity and a range of child mental health outcomes (parent-reported) and psychosocial outcomes (child-reported) at age 7–8 years. This study addresses limitations of previous studies by using latent classes that describe patterns of maternal religious belief (Halstead et al., 2022) measured before the birth of the child. In the present study the latent classes describe qualitatively distinct patterns of religious belief and distinguish between highly religious, moderately religious, Agnostic and Atheist. The analysis adjusts for a range of confounders including parental mental health, parental adverse childhood events, demographic variables, and SEP indicators.

We have the following research objectives:

1. Examine the risk ratios of each religious latent class for the following mental health symptoms – Attention deficit hyperactivity disorder (ADHD), conduct disorder, depression, general anxiety, obsessive compulsive disorder (OCD), social and specific phobias, oppostional defiant disorder (ODD), and separation anxiety using logistic regression in relation to a reference class taken from a latent class analysis of maternal religious beliefs.
2. Examine the risk ratios of each religious latent class for the following psychosocial outcomes – antisocial behaviour, low scholastic competence, low self-worth, victim of overt bullying, overt bully, relational bully, victim of relational bullying, and unhappiness with friends, using logistic regression in relation to a reference class taken from a latent class analysis of maternal religious beliefs.

## Methods

### Participants

**Figure 1.**
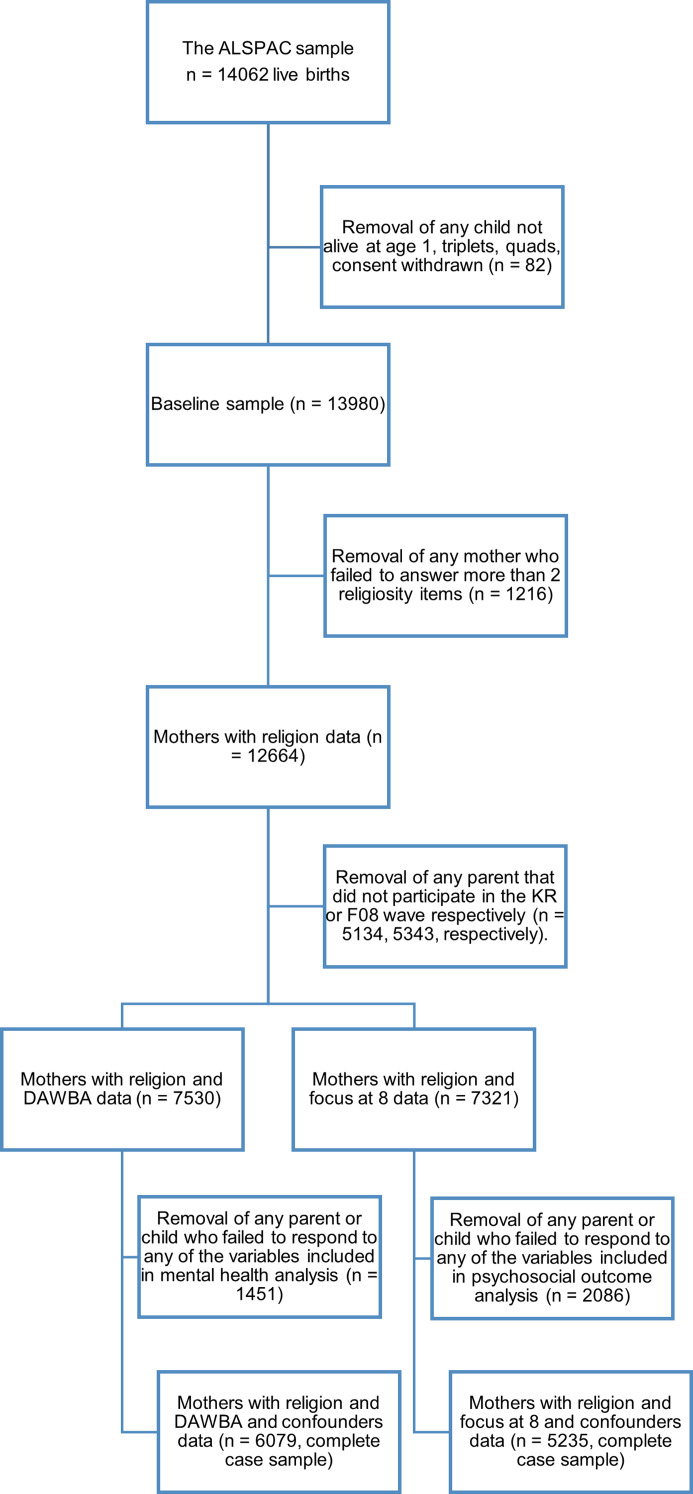
Sample flowchart showing each stage of exclusion.

The ALSPAC was established to understand how genetic and environmental characteristics influence health and development in parents and children. All pregnant women resident in a defined area in the Southwest of England, with an expected date of delivery between 1 April 1991 and 3 December 1992 were invited to take part in the study. The initial number of pregnancies enrolled is 14 541. Of these initial pregnancies, there was a total of 14 676 foetuses, resulting in 14 062 live births and 13 988 children who were alive at 1 year of age. These parents and children have been followed over the last 30 years and have completed a variety of questionnaires concerning their demographics, physiological and genetic data, life events, physical, and psychological characteristics. For more information, see Boyd et al., 2013; Fraser et al., 2013. Please note that the study website contains details of all the data that is available through a fully searchable data dictionary and variable search tool (http://www.bristol.ac.uk/alspac/researchers/our-data/). Overall, our sample was 97.4% White at baseline, 64.4% identified as Church of England, 15.3% as No religion, 8.3% as Roman Catholic, 7.8% as Other Christian and the remainder as a non-Christian religious group (Fig. 1).

## Exposures

### Latent classes of maternal religiosity

Latent class analysis is a ‘person-centred’ statistical technique (Nylund-Gibson & Choi, 2018) that uses a set of observed variables and the conditional probability of responding in a particular pattern to those variables to probabilistically assign participants to a mutually exclusive unobserved group (i.e. latent class).We used the maternal latent class membership variables derived by Halstead et al. (2022) as our indicators of religiosity in the mothers of the ALSPAC parent cohort measured in 1990, based upon the assumptions that they would be the primary caregiver, the classes are stable over time (Major-Smith et al., 2023) and that maternal and paternal religious latent classes are associated (see Halstead et al., 2022). The latent classes are composed of a series of conditional probabilities, which are used to label and describe the classes. These classes provide a more nuanced alternative to variable centred approaches that measure religiosity using a single item by providing qualitatively distinct types of religious belief rather than a simple continuum. The questions used to derive the classes include belief in God, whether a person has asked for help from God, whether they would ask for God's help when in trouble, the duration of their faith, their church attendance, and whether they have received help from individuals from their own or another religion, which were measured at the antenatal timepoint. Our choice to use the latent classes generated at the antenatal (1990) timepoint were motivated by the larger sample size provided by this timepoint. This decision is also supported by the small amount of transition between classes over time (see Major-Smith et al., 2023 for a descriptive account of these transitions). The classes were named the Highly Religious, Moderately Religious, Agnostic, and Atheist and each represented approximately 14, 30, 38 and 18% of the sample, respectively. The Highly Religious class exhibits a consistent endorsement of religious beliefs, high level of church attendance, and high likelihood to obtain help and support from religious individuals. The Moderately Religious class shares many of the characteristics of the Highly Religious but were highly unlikely to visit church regularly. The Agnostic class expressed an uncertainty about the existence of God and were also uncertain about whether they would ask for help from God if they were in trouble or whether God had helped them previously. The Atheists possessed strong disbelief in the existence of God, and general disagreement with statements related to religious belief and practice. For details of the questions used to derive the latent classes and the conditional probabilities of each class, see Table S2 and S3 of the appendix, respectively.

## Outcomes

### Parent-reported child mental health outcomes

When their study child was aged 7 years (approximately 1997/8), mothers were asked to complete the Development and Wellbeing Assessment (DAWBA) (Goodman, Ford, Richards, Gatward, & Meltzer, 2000) which includes questions about symptoms of common mental health disorders. This was based upon the rationale that age 7/8 are associated with mental health problems in adolescence and adulthood (Fergusson, John Horwood, & Ridder, 2005), and we are looking at the associations with maternal RSBB, and maternal factors have a stronger influence in childhood than in adolescence (Bhargava & Witherspoon, 2015; Lau, Faulkner, & Qian, 2016; Powell, Son, File, & Froiland, 2012). Later timepoints will be explored as part of this programme of research. We included symptoms of separation anxiety, phobias, social anxiety, OCD, generalised anxiety disorder, depression, ADHD, ODD and conduct disorder. A small number of children met DSM-IV criteria for psychiatric disorders in the ALSPAC cohort at this age (Joinson, Heron, Emond, & Butler, 2006). We therefore created binary variables to indicate the presence of any symptom that was severe, e.g., rated as ‘a lot more than others’ or ‘a great deal’ (See Table 1 of the appendix for details of items used). Only the complete case samples were used for analyses. The prevalence of mental health symptoms is provided in Table S4 of the appendix.

**Table 1.** Distribution of confounders across the samples

Sample size		Baseline (up to 13 980)	Mothers with religion data (12 664)	Religion and DAWBA (7530)	Religion and DAWBA and confounders (6079)	Religion and focus at 8 (7321)	Religion and Focus at 8 and confounders (5235)
Social class (Ref; non-manual)	Manual	2933 (25.6%)	2611 (24.6%)	1401 (21.1%)	1235 (20.3%)	1353 (21.0%)	1038 (19.8%)
Financial problems (Ref; no)	Yes	1510 (12.1%)	1319 (11.6%)	665 (9.6%)	569 (9.4%)	673 (10.0%)	510 (9.7%)
Financial hardship (Ref; no)	Yes	3298 (26.1%)	2970 (25.3%)	1590 (22.0%)	1265 (20.8%)	1494 (21.3%)	1026 (19.9%)
Education (Ref; A-level or higher)	None/vocational	3869 (29.8%)	3381 (28.2%)	1621 (21.9%)	1259 (20.7%)	1524 (21.3%)	1019 (19.5%)
	O Level	4485 (34.6%)	4243 (35.4%)	2648 (35.8%)	2228 (36.7%)	2528 (35.3%)	1875 (35.8%)
Home status (Ref; bought)	Rented	3578 (26.4%)	2941 (24.0%)	1282 (17.4%)	893 (14.7%)	1136 (15.8%)	695 (13.3%)
ACE (Ref; 0 ACE)	1	2026 (20.4%)	2330 (18.4%)	1479 (19.6%)	1174 (19.3%)	1424 (19.5%)	988 (18.7%)
	2	1387 (14.0%)	1419 (11.2%)	860 (11.4%)	684 (11.3%)	813 (11.1%)	573 (10.8%)
Maternal age at baseline	Mean (s.d.)	28.4 (4.8)	28.5 (4.7)	29.1 (4.5)	29.3 (4.4)	29.3 (4.5)	29.5 (4.4)
Maternal depression	Median (IQR)	6 (7)	6 (7)	6 (6)	6 (6)	6 (7)	6 (6)
Maternal anxiety	Median (IQR)	4 (5)	4 (5)	4 (5)	4 (5)	4 (5)	4 (4)

*Note*. The samples in the table correspond to the samples in the flowchart.

### Self-reported psychosocial outcomes

Child self-reported psychosocial outcomes were obtained from a ‘Focus Clinic’ attended by children when they were aged 8 years. The prevalence of psychosocial outcomes is provided Table S5 of the appendix. All reported Cronbach's *α* refer to the current paper, using the complete case samples.

#### Bullying (as the bully and victim)

We assessed peer victimisation through self-report using a modified version of the bullying and friendship interview schedule (Wolke, Woods, Stanford, & Schulz, 2001). The scale consists of measures of being an overt victim (*α* = 0.593), relational victim (*α* = 0.623), overt bully (*α* = 0.510), and relational bully (*α* = 0.447).

#### Scholastic competence and global self-worth

This measure used a 12-item version of Harter's Self Perception Profile for Children (Harter, 1985) comprising global self-worth (*α* = 0. 651) and scholastic competence (*α* = 0. 691), rather than the full 36 item scale that contains the other subscales.

#### Unhappy with friends

A series of five questions taken from the Cambridge Hormones and Moods Project Friendship Questionnaire (Goodyer, Wright, & Altham, 1990) to indicate unhappiness with friends (*α* = 0. 503).

#### Antisocial activities

This measure used 15 questions, including 11 from the Self-Reported Antisocial Behaviour for Young Children Questionnaire (Loeber, Stouthamer-Loeber, Van Kammen, & Farrington, 1989), three dummy questions and an additional example question, to measure antisocial activities (*α* = 0. 582).

### Confounders

We chose confounders based upon empirical evidence of a relationship with the exposure and outcome variables. Confounders were assessed by maternal reports in the antenatal period and included maternal age at baseline, maternal mental health, and SEP indicators. We also adjusted for retrospective reports of maternal ACEs assessed in questionnaires completed during the antenatal period and when the study child was aged 2 years. Details of for the construction of the confounding variables are provided in Table S1 of the appendix.

## Statistical analyses

We used logistic regression to calculate odds ratios and 95% confidence intervals for each mental health and psychosocial variable and their association with maternal religious latent class, both before and after adjustment for confounders. As the outcomes we are examining are rare (⪅10%) we interpreted the effect estimates as risk ratios, and we attributed changes seen to parameter estimates in the multivariable models as being due to confounding (Zammit, Allebeck, Andreasson, Lundberg, & Lewis, 2002). Risk ratios were estimated in relation to the Agnostic class, as this was the largest class in the sample, and was characterised by the most ‘neutral’ beliefs (i.e. neither religious nor Atheist) (Johfre & Freese, 2021).Parameter estimates were then adjusted for confounders. The dataset was constructed in R studio (R Core Team, 2021) and all analyses were carried out in Mplus (Version 8.7), using a bias adjusted 3 step latent class analysis which incorporates uncertainty in latent class assignment (Heron, Croudace, Barker, & Tilling, 2015; Vermunt, 2010; Vermunt & Magidson, 2021).

### Weighted estimates

To address potential bias due to missing data, we created weighting variables based on SEP variables as SEP is associated with attrition in the ALSPAC sample (Fernández-Sanlés et al., 2021; Howe, Tilling, Galobardes, & Lawlor, 2013). This was done by using inverse probability weighting, using SEP indicators with minimal missingness (less than 5%) including home ownership status, cigarette smoking, car ownership, and education, with any missingness recoded in these variables to be the modal response category. These were then used as exposures in a logistic regression model, with missingness at the 7-year and 8-year timepoints (when our outcome variables were measured) as the outcome variables. From these models, the weights were calculated and added to the main dataset, for use in the weighted analyses. The results of the weighted analyses are presented as the main results and the unweighted results are provided in Table S8 and S9 of the appendix. Additionally, full details of each adjusted model may be found in Table S6 and S7 of the appendix.

## Results

Compared with the baseline sample, the restricted sample used in the complete case analysis comprised a higher proportion of participants of higher SEP (i.e. homeowners, non-manual social class, no major financial problems, and no financial hardship). The restricted sample also had a lower proportion of maternal depression, anxiety and adverse childhood events. See Table 1 for more details.

### Association between maternal religiosity latent classes and parent-reported child mental health outcomes

Table 2 shows the results of the logistic regressions with parent-reported mental health variables as outcomes. In the unadjusted models, children of mothers in the **Highly Religious** class**,** compared with the Agnostic class have increased risk of ADHD, depression, OCD, and ODD. Compared with the Agnostic class, children of mothers in the **Moderately Religious** class have increased risk of depression, anxiety, and ODD. Compared with the Agnostic class, children of mothers in the **Atheist** class have increased risk of ADHD, and conduct disorder. The highest level of attenuation in the adjusted models was found for conduct disorder, with a 9% reduction in the risk ratio in the fully adjusted model. There was little evidence of confounding in the adjusted models for the other mental health symptoms.

**Table 2.** Weighted risk ratios and 95% confidence intervals for the associations between maternal religious latent class and offspring mental health at age 7, with the Agnostic class as the reference class

	OR (CI) Model 1	OR (CI) Model 5		OR (CI) Model 1	OR (CI) Model 5
**ADHD**			**Social phobia**		
Agnostic	1.00 ref	1.00 ref	Agnostic	1.00 ref	1.00 ref
Highly religious	1.29 (0.97–1.73)	1.34 (1.00–1.80)	Highly religious	1.39 (0.96–2.01)	1.33 (0.91–1.95)
Moderately religious	1.16 (0.90–1.49)	1.17 (0.91–1.52)	Moderately religious	1.25 (0.89–1.75)	1.24 (0.88–1.73)
Atheist	1.44 (1.11–1.88)	1.41 (1.08–1.85)	Atheist	1.04 (0.71–1.53)	1.01 (0.68–1.49)
*p* value	0.043	0.053	*p* value	0.255	0.328
**Conduct disorder**			**Specific phobia**		
Agnostic	1.00 ref	1.00 ref	Agnostic	1.00 ref	1.00 ref
Highly religious	1.35 (0.94–1.96)	1.37 (0.94–2.00)	Highly religious	1.01 (0.77–1.32)	1.06 (0.80–1.39)
Moderately religious	1.19 (0.86–1.66)	1.22 (0.87–1.71)	Moderately religious	1.03 (0.82–1.29)	1.05 (0.83–1.32)
Atheist	1.55 (1.11–2.18)	1.46 (1.04–2.05)	Atheist	0.86 (0.66–1.12)	0.84 (0.65–1.10)
*p* value	0.067	0.133	*p* value	0.585	0.383
**Depression**			**ODD**		
Agnostic	1.00 ref	1.00 ref	Agnostic	1.00 ref	1.00 ref
Highly religious	1.51 (1.15–1.97)	1.40 (1.07–1.85)	Highly religious	1.73 (1.21–2.49)	1.72 (1.19–2.48)
Moderately religious	1.50 (1.19–1.90)	1.48 (1.17–1.87)	Moderately religious	1.39 (1.00–1.94)	1.38 (0.99–1.94)
Atheist	1.17 (0.89–1.53)	1.13 (0.86–1.48)	Atheist	1.44 (1.00–2.06)	1.39 (0.96–1.99)
*p* value	0.001	0.004	*p* value	0.020	0.027
					
**General anxiety**			**Separation anxiety**		
Agnostic	1.00 ref	1.00 ref	Agnostic	1.00 ref	1.00 ref
Highly religious	1.21 (0.88–1.66)	1.22 (0.88–1.69)	Highly religious	1.19 (0.83–1.71)	1.18 (0.81–1.72)
Moderately religious	1.44 (1.11–1.88)	1.43 (1.10–1.87)	Moderately religious	1.23 (0.91–1.68)	1.25 (0.92–1.71)
Atheist	1.24 (0.92–1.67)	1.24 (0.92–1.67)	Atheist	1.09 (0.77–1.54)	1.05 (0.74–1.48)
*p* value	0.056	0.066	*p* value	0.552	0.775
**OCD**					
Agnostic	1.00 ref	1.00 ref			
Highly religious	1.60 (1.08–2.37)	1.53 (1.03–2.26)			
Moderately religious	0.65 (0.41–1.03)	0.64 (0.41–1.02)			
Atheist	1.04 (0.69–1.59)	1.03 (0.68–1.57)			
*p* value	0.004	0.008			

*Note. Model 1* is the unadjusted model, *Model 5* adjusts for maternal age, SEP, ACE, and maternal mental health. *p* values are omnibus *p* values based on a Wald test with 3 df.

### Association between maternal religiosity latent classes and self-reported psychosocial outcomes

Table 3 that compared with the Agnostic class, children of mothers in the **Highly Religious** class have increased risk of antisocial behaviour, and being the victim of relational bullying and decreased risk of being an overt bully. Compared with the Agnostic class, children of mothers in the **Moderately Religious** class have increased risk of self-reported antisocial behaviour and low scholastic competence. Compared with the Agnostic class, children of mothers in the **Atheist** class have increased risk of self-reported antisocial behaviour, but lower risk of being overt bullies and being unhappy with friends. The highest level of attenuation in the adjusted models was found for low scholastic competence, with an 8% increase in the risk ratio in the fully adjusted model. There was little evidence of confounding in the adjusted models for the other psychosocial outcomes.

**Table 3.** Weighted risk ratios and 95% confidence intervals for the associations between maternal religious latent class and offspring psychosocial outcomes at age 8, with the Agnostic class as the reference class

	OR (CI) Model 1	OR (CI) Model 5		OR (CI) Model 1	OR (CI) Model 5
**Antisocial behaviour**			**Overt bully**		
Agnostic	1.00 ref	1.00 ref	Agnostic	1.00 ref	1.00 ref
Highly religious	1.29 (1.03–1.62)	1.29 (1.02–1.62)	Highly religious	0.60 (0.41–0.88)	0.59 (0.40–0.87)
Moderately religious	1.24 (1.02–1.51)	1.24 (1.01–1.51)	Moderately religious	0.74 (0.52–1.05)	0.73 (0.51–1.04)
Atheist	1.42 (1.15–1.77)	1.40 (1.12–1.73)	Atheist	0.57 (0.40–0.83)	0.58 (0.40–0.84)
*p* value	0.008	0.013	*p* value	0.013	0.013
**Low scholastic competence**			**Relational bully**		
Agnostic	1.00 ref	1.00 ref	Agnostic	1.00 ref	1.00 ref
Highly religious	0.96 (0.77–1.19)	1.04 (0.84–1.30)	Highly religious	0.71 (0.40–1.27)	0.65 (0.36–1.17)
Moderately religious	1.17 (0.98–1.40)	1.22 (1.02–1.46)	Moderately religious	1.03 (0.59–1.80)	1.05 (0.60–1.84)
Atheist	1.10 (0.90–1.35)	1.12 (0.91–1.37)	Atheist	0.90 (0.50–1.63)	0.90 (0.50–1.63)
*p* value	0.241	0.184	*p* value	0.642	0.458
**Low self-worth**			**Victim of relational bullying**		
Agnostic	1.00 ref	1.00 ref	Agnostic	1.00 ref	1.00 ref
Highly religious	1.26 (0.96–1.64)	1.27 (0.97–1.67)	Highly religious	0.77 (0.60–0.99)	0.74 (0.57–0.95)
Moderately religious	1.15 (0.91–1.45)	1.17 (0.93–1.49)	Moderately religious	0.94 (0.75–1.17)	0.92 (0.74–1.15)
Atheist	1.17 (0.91–1.52)	1.16 (0.89–1.50)	Atheist	1.08 (0.84–1.39)	1.09 (0.84–1.41)
*p* value	0.324	0.289	*p* value	0.103	0.045
**Victim of overt bullying**			**Unhappy with friends**		
Agnostic	1.00 ref	1.00 ref	Agnostic	1.00 ref	1.00 ref
Highly religious	0.91 (0.75–1.12)	0.89 (0.73–1.10)	Highly religious	1.15 (0.91–1.47)	1.15 (0.91–1.47)
Moderately religious	0.95 (0.80–1.13)	0.95 (0.80–1.13)	Moderately religious	0.93 (0.75–1.15)	0.94 (0.76–1.16)
Atheist	0.94 (0.78–1.14)	0.96 (0.79–1.16)	Atheist	0.77 (0.60–0.98)	0.76 (0.59–0.98)
*p* value	0.827	0.743	*p* value	0.047	0.043

*Note*. *Model 1* is the unadjusted model, *Model 5* adjusts for maternal age, SEP, ACE, and maternal mental health. *p* values are omnibus *p* values based on a Wald test with 3 df.

## Discussion

The current study found evidence that maternal religious belief was associated with a range of mental health and psychosocial outcomes in their offspring at age 7–8 years. Compared with children of Agnostic mothers, the children of Highly religious and Moderately religious parents were at greater risk of internalising symptoms, and the children of Atheist parents were at greater risk of externalising symptoms. However, there was no clear pattern of results for psychosocial outcomes. These associations were independent of maternal age, SEP variables, ACE, and mental health. There were only a few instances of attenuation, with most of the results being robust to the inclusion of confounding variables.

### Strengths and limitations

There are several strengths to this study. The use of data from a large prospective community based cohort, use of latent classes of belief, rather than relying upon single item measures which dominate the religiosity literature, availability of parent- and child-reported mental health and psychosocial outcomes based on validated questionnaires, and availability of data on a wide range of important confounders The use of latent classes provided insights that may have otherwise been lost by using items that fail to differentiate between Agnostic and Atheist individuals, such as through the use of items that ask participants to indicate the importance of religion in their lives or church attendance.

There are also limitations that should be considered when interpreting the findings. There was a large amount of attrition between the baseline sample, and the final complete case analysis which could lead to selection bias. Specifically, those with higher SEP (Howe et al., 2013) and religiosity (Morgan, Halstead, Northstone, & Major-Smith, 2022) are more likely to participate in ALSPAC initially and continue to participate over time. Attrition is strongly related to low SEP in ALSPAC, and the present study attempted to mitigate this using weighting to account for the potential bias, in line with recommendations (Howe et al., 2013). Our study is also limited in its generalisability, given the very small numbers of Non-White, and Non-Christian religious individuals, which prevents us from generalising to these groups. Furthermore, due to the data on religious belief and mental health being collected in the 90s, there is the possibility of cohort effects, as well as the changes in prevalence and perception of religion over time. These factors may mean that our findings may not generalise to the present day. Finally, it is possible that the concordance/discordance between maternal and paternal religious classes may play a role in offspring mental health outcomes (van der Jagt-Jelsma et al., 2011). This possibility should be explored in future research.

### Comparisons with previous research

Our findings are contrary to previous research which examined the relationship between parental religious belief and child mental health, which has found that greater parental (either maternal or paternal) religious belief is associated with better mental health outcomes in offspring (Svob et al., 2018; Varon & Riley, 1999). A possible reason for this difference is that the current study is based on a UK sample, compared to previous research which has predominantly been based on US samples (e.g. 75% of the religious belief and mental health research reviewed by Koenig, 2009 was conducted on US samples). There is evidence the UK differs in its relationship with religious belief and mental health compared to other countries, with some studies finding that increased religious or spiritual belief was associated with worse mental health outcomes such as depression, anxiety, and phobias (King et al., 2013; Leurent et al., 2013). Additionally, given that the US is highly religious compared to the UK, and most previous studies contain relatively small numbers of individuals that could be considered Atheist or Agnostic, previous studies may be less suited to capturing the differences between them and religious individuals.

The use of items that assume religious belief exists on a simple continuum from non-religious to highly religious may be obscuring the true nature of religion's relationship with mental health and psychosocial outcomes. The relationship between religious belief and mental health may be better explained by using qualitatively different types of belief in future research. Given previous literature's inclination to use items that ask about religious attendance or the importance of religion, and both Atheists and Agnostics are likely to respond the same way to these items (i.e. they are both unlikely to attend church), previous research may be failing to acknowledge the way these items function for different groups. Conflating Atheist and Agnostic groups appears to hide important differences in outcomes between the two.

### Possible mechanisms explaining the current findings

The increased mother-reported externalising problems and psychosocial outcomes in the Highly Religious mothers (e.g. ADHD, ODD, antisocial behaviour) may be partially explained by highly religious mothers having higher expectations of their child's morals (Rhodes & Nam, 1970; Smith, 2003), an increased likelihood to perform parental monitoring activities (Guo, 2018; Kim & Wilcox, 2014) and to be more engaged in their child's life (Guo, 2018). This possibly leads them to be more attentive to ADHD or ODD symptoms, as well as negative (e.g. antisocial) behaviours. However, children of religious parents also perceive their parents to be more controlling, which could in turn lead to more internalising and externalising problems (Bornstein et al., 2017). Combined, this suggests that a degree of monitoring or control is healthy and may lead to more attentive parenting, but when it is perceived to be excessive, it can be a stressor to the child. In the context of the current study, the higher degree of control that religious mothers exert over their children may impact their levels of stress, leading to a greater risk of depression or anxiety (Rapee, 1997; Yap, Pilkington, Ryan, & Jorm, 2014). The increased mother reported internalising symptoms may be explained by children of religious parents being religious themselves, which may incline them towards rumination, which leads to greater internalising symptoms (Saunders et al., 2021). Religious belief is associated with OCD (Abramowitz, Deacon, Woods, & Tolin, 2004; Himle, Chatters, Taylor, & Nguyen, 2011; Sica, Novara, & Sanavio, 2002), possibly through heightened pathogen disgust sensitivity (Olatunji et al., 2007), which is also associated with religiosity (Stewart, Adams, & Senior, 2020; Yu, Bali, Tsikandilakis, & Tong, 2022). Consequently, the behaviours associated with OCD symptoms may be taught to offspring during their upbringing (Waters & Barrett, 2000). There is little existing literature to explain the pattern of results in the Atheist class, or for the differences between the Moderately and Highly Religious classes. We may nevertheless speculate that the association between spirituality, depression, and other internalising disorders may be partially explained by an increased interior-orientation, observed for example in rumination (Saunders et al., 2021) and default-mode network connectivity (Svob, Wang, Weissman, Wickramaratne, & Posner, 2016) in those who are spiritual. Whereas Atheism may support a more external worldview and contribute to its greater likelihood to be associated with externalising disorders. Further research is needed to examine the replicability of our findings in non-US samples.

## Conclusions

Contrary to existing literature, we found evidence that maternal religiosity is associated with a higher risk of internalising symptoms. Children of Atheist parents are at greater risk of externalising symptoms. Future research is needed to determine whether these relationships are causal and to identify the underlying mechanisms. For example, it is conceivable that the relationship may be mediated by parenting style/quality variables or moderated by partner religious class.

## Supporting information

Halstead et al. supplementary material Halstead et al. supplementary material

## Data Availability

Please see the ALSPAC data management plan which describes the policy regarding data sharing (http://www.bristol.ac.uk/alspac/researchers/data-access/documents/alspac-data-management-plan.pdf), which is by a system of managed open access. Data used for this submission will be made available on request to the Executive (alspac-exec@bristol.ac.uk). The datasets presented in this article are linked to ALSPAC project number B4001, please quote this project number during your application. The steps below highlight how to apply for access to the data included in this study and all other ALSPAC data:
Please read the ALSPAC access policy (http://www.bristol.ac.uk/media-library/sites/alspac/documents/researchers/data-access/ALSPAC_Access_Policy.pdf) which describes the process of accessing the data and samples in detail, and outlines the costs associated with doing so. You may also find it useful to browse our fully searchable research proposals database (https://proposals.epi.bristol.ac.uk/?q=proposalSummaries), which lists all research projects that have been approved since April 2011. Please submit your research proposal (https://proposals.epi.bristol.ac.uk/) for consideration by the ALSPAC Executive Committee. You will receive a response within 10 working days to advise you whether your proposal has been approved.

## Supplementary material

The supplementary material for this article can be found at https://doi.org/10.1017/S003329172300079X.
